# Supplementary material for: Selective suppression of melanoma lacking IFN-γ pathway by JAK inhibition depends on T cells and host TNF signaling
Source: Nat Commun. 2022 Aug 25;13:5013. doi: 10.1038/s41467-022-32754-7 (PMC9411168; doi:10.1038/s41467-022-32754-7)
Supplement: Supplementary file 1 — Supplementary Information [file 41467_2022_32754_MOESM1_ESM.pdf]

Supplementary Figure 1. Characterization of IFNγR1^{KD} and IFNγR1^{KO} melanoma cells.

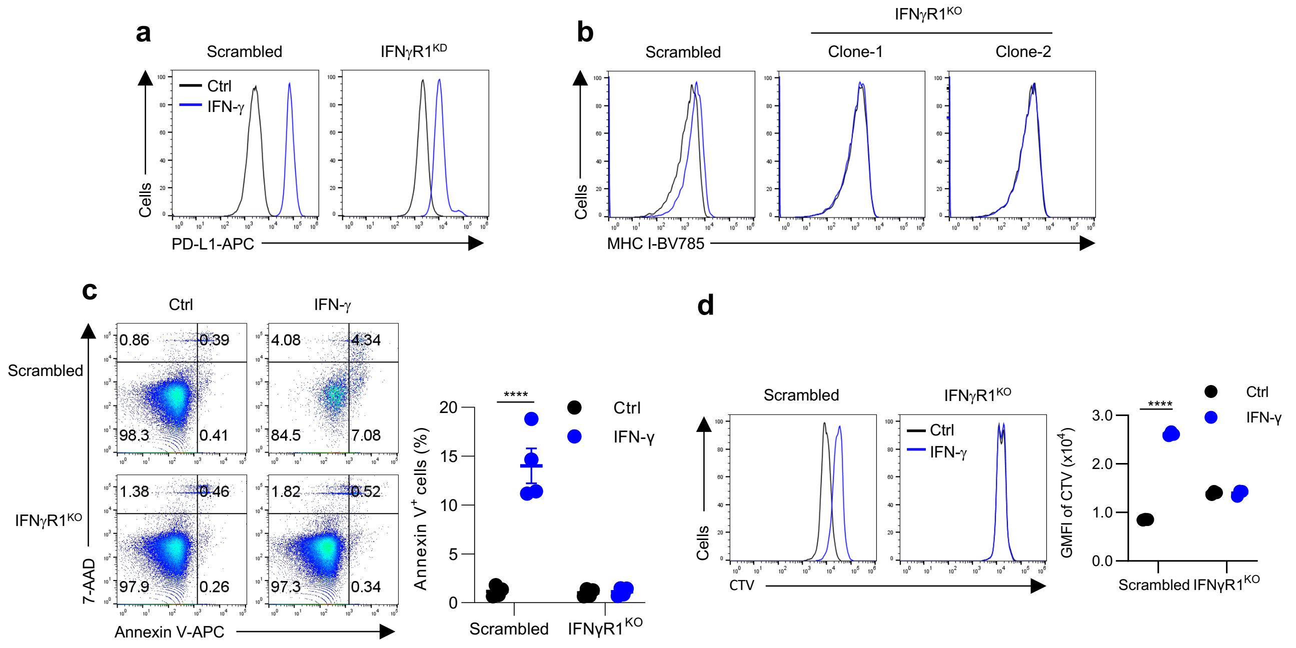

a. PD-L1 expression in scrambled control and IFNγR1^{KD} cells stimulated with or without (Ctrl) 100 U/mL IFN-γ for 24h. **b**. MHC I expression in scrambled and IFNγR1^{KO} cells stimulated with or without (Ctrl) 100 U/mL IFN-γ for 24h. Representative results from 3 independent experiments were shown. **c.** Cell death of scrambled and IFNγR1^{KO} cells treated with or without (Ctrl) 100 U/mL IFN-γ for 96h, analyzed by Annexin V and 7-AAD staining (n=4 per group). **d.** CellTrace Violet (CTV)-labeled scrambled and IFNγR1^{KO} cells were similarly treated as in **c** and CTV dilution (a readout of cell proliferation) was shown (n=4 per group). All were done by flow cytometry (FACS strategy 1). Representative data in **c**-**d** from 3 independent experiments were depicted as means ± SEM in scatter plots. ****, p=0.00008 for **c,** and ****, p=0.00002 for **d,** by two-way ANOVA with Šídák's multiple comparisons test with adjustment. Source data are provided as a Source Data file.

Supplementary Figure 2. Reduced effector function of TILs from IFNγR1^{KO} melanomas and decreased survival probability of patients with IFNGR1^{Low} SKCMs.

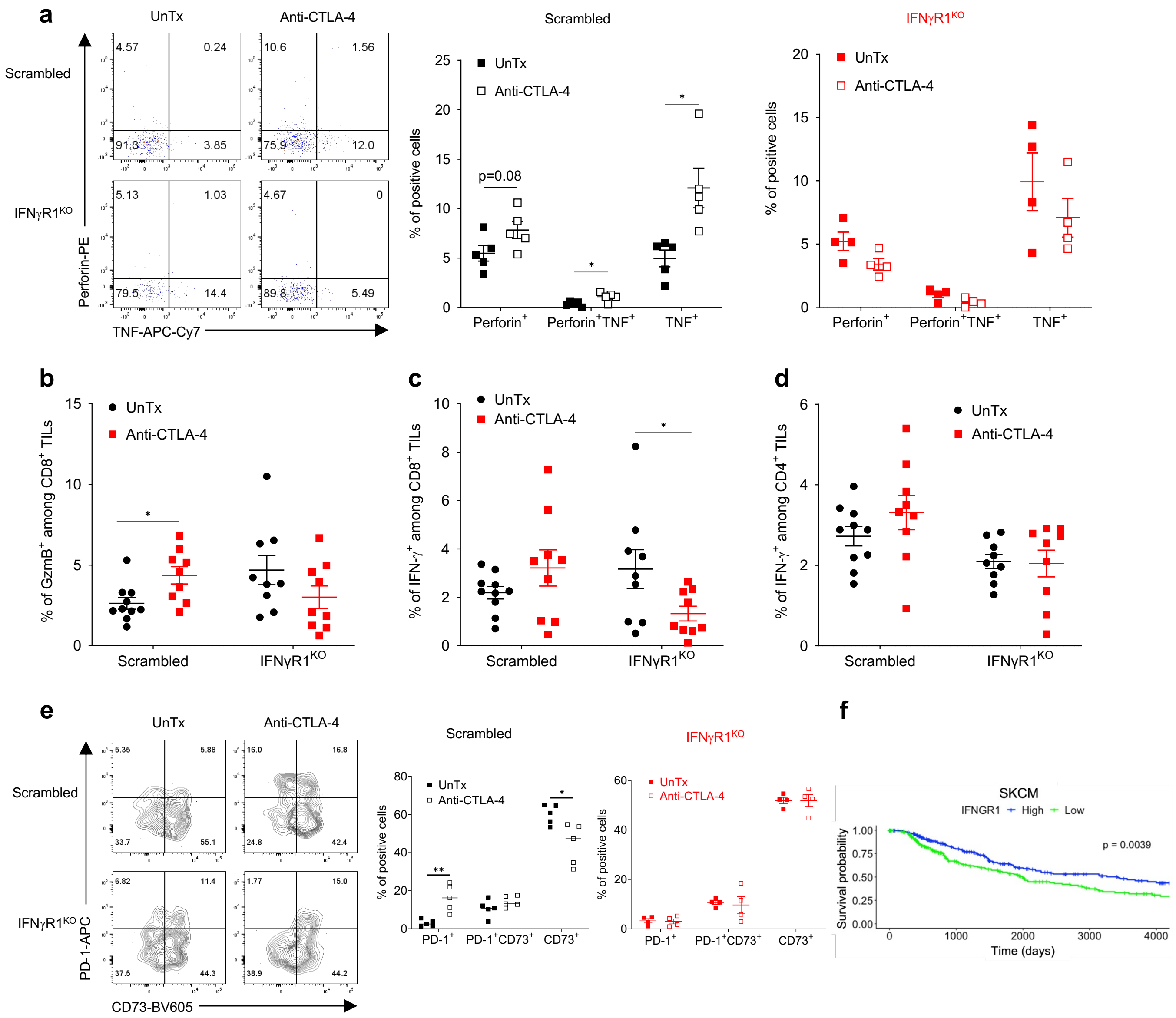

Mice bearing scrambled or IFNγR1^{KO} melanomas were treated with anti-CTLA-4 or isotype control (UnTx). Isolated tumor-infiltrating lymphocytes (TILs) were analyzed for production of perforin and TNF by CD4^{+} TILs (**a**) (*, p=0.0136 for Perforin^{+}TNF^{+}; *, p=0.0115 for TNF^{+}), of GzmB by CD8^{+} TILs (**b**) (*, p=0.0139), and of IFN-γ by CD8^{+} (**c**) (*, p=0.0481) and CD4^{+} TILs (**d**) after a brief stimulation with PMA and ionomycin. **e**. Surface expression of PD-1 and CD73 of unstimulated CD4^{+} TILs. *, p=0.0185; **, p=0.0033. Two-sided Student's t-test was used in **a-e** for statistical analyses. N=5 for Scrambled UnTx/Anti-CTLA-4; n=4 for IFNγR1^{KO} UnTx/Anti-CTLA-4 in **a** and **e**. N=10 for Scrambled UnTx; n=9 for Scrambled Anti-CTLA-4, IFNγR1^{KO} UnTx and IFNγR1^{KO} Anti-CTLA-4 group in **b**, **c** and **d**. FACS strategy 3 was applied for **a-e.** Representative data from 2 independent experiments were presented as means ± SEM in scatter plots. **f**. Survival probability of patients with IFNGR1^{High} (n=234) vs IFNGR1^{Low} (n=235) SKCMs in TCGA database. The log-rank test was used for statistical analysis (p=0.0039). Source data are provided as a Source Data file.

Supplementary Figure 3. Active protein tyrosine kinases in IFNγR1^{KD} and IFNγR1^{KO} cells.

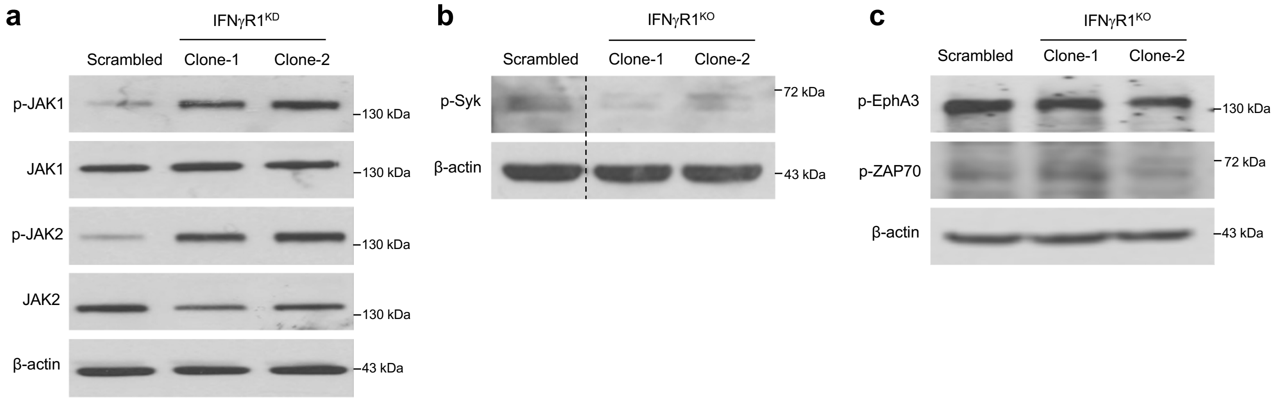

Western blot analysis of p-JAK1/2 and total JAK1/2 in scrambled and IFNγR1^{KD} cells (**a**), of p-Syk (Tyr526) (**b**) as well as p-EphA3 (Tyr779) and p-ZAP-70 (Tyr493) (**c**) in scrambled and IFNγR1^{KO} cells. β-actin was used as a loading control. Experiments were repeated twice (**c**) or thrice (**a, b**) with similar results. Source data are provided as a Source Data file.

Supplementary Figure 4. Responses of IFNγR1^{KO} cells to the blockade of IL-6 and IL-6R and IFN-α stimulation.

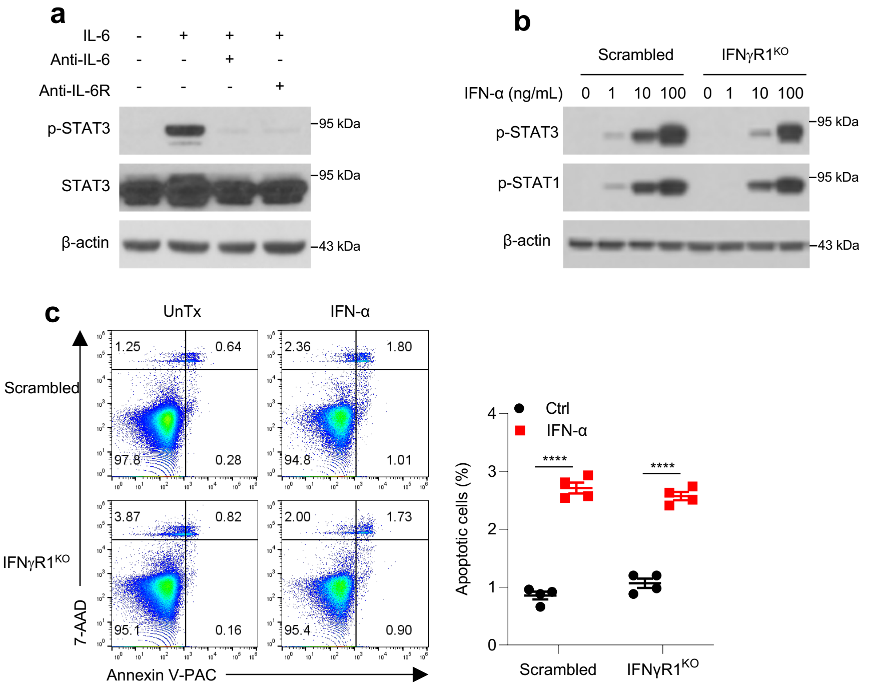

a. B16-BL6 cells were treated with vehicle (UnTx) or 100 ng/mL IL-6 for 48h ± blocking antibodies against IL-6 (10 μg/mL) or IL-6R (10 μg/mL), followed by analyses of p-STAT3 and total STAT3 by Western blot (WB). β-actin was used as a loading control. **b-c**. Scrambled and IFNγR1^{KO} cells were pretreated with various concentrations of IFN-α for 15 min to analyze p-STAT3 and p-STAT1 by WB (**b**) or treated with 100 ng/mL IFN-α for 96h to evaluate cell death by flow cytometric analyses of Annexin-V/7-AAD (**c**) (n=4 per group) (FACS strategy 1). Representative results shown in the scatter plot were means ± SEM. ****, p=0.00002 (left); ****, p=0.00004 (right), by two-way ANOVA with Šídák's multiple comparisons test (with adjustment). Experiments were repeated thrice with similar results. Source data are provided as a Source Data file.

Supplementary Figure 5. p-4E-BP1 in IFNγR1^{KO} cells and expression of mTOR and JAK1/2 target genes in human melanomas.

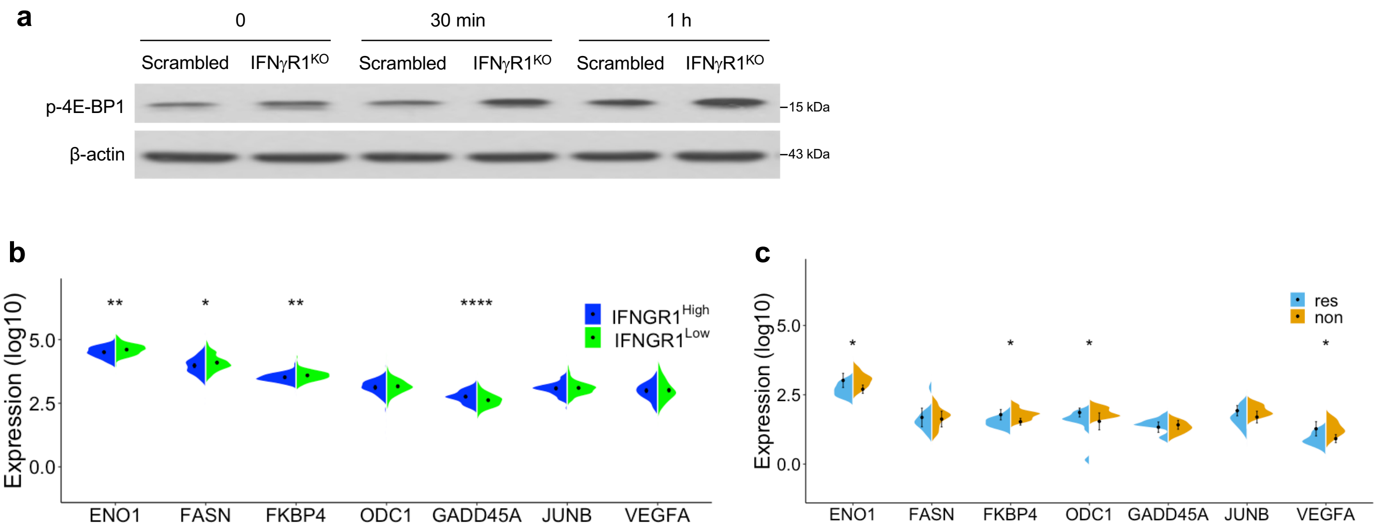

a. p-4E-BP1 in scrambled control and IFNγR1^{KO} cells treated with 10 μM Ruxo for designated times, analyzed by Western blot. β-actin was used as a loading control. Experiments were repeated twice with similar results. **b**. Expression of indicated genes in IFNGR1^{High} (n=96) vs IFNGR1^{Low} (n=155) SKCMs from the TCGA database after deconvolution. **c**. Expression of indicated genes in anti-PD-1-responsive (res, n=12) vs non-responsive (non, n=7) melanomas in GSE78220 after deconvolution. Violin plots showed the distribution of normalized expression (log10) of genes. The center points denote median values. The comparisons in **b** and **c** were done using Mann-Whitney U test. In **b**, *, p=0.0166, **p=0.0018 for ENO1, **p=0.0051 for FKBP4, ****p=0.00008. In **c**, *, p=0.0221 for ENO1, *p=0.0221 for FKBP4, *p=0.0358 for ODC1, *p=0.0171 for VEGFA. Source data are provided as a Source Data file.

Supplementary Figure 6. Selective suppression of IFNγR1^{KO} melanomas by Ruxo.

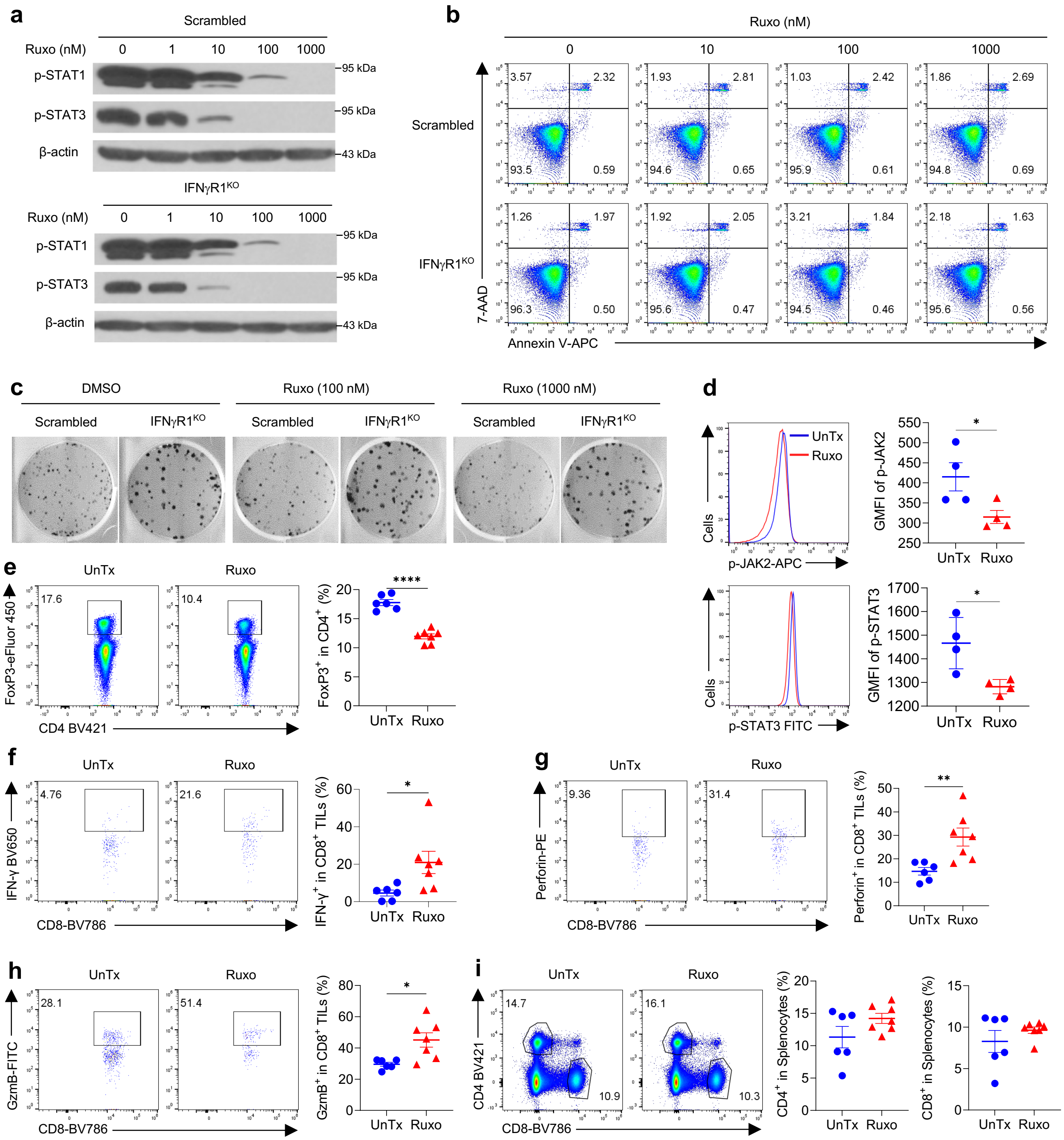

a. p-STAT1/3 in scrambled control and IFNγR1^{KO} cells treated with Ruxo for 2.5h and 10 ng/mL IFN-α for the last 15 min. **b.** Apoptosis of cells treated ± Ruxo for 72h, analyzed with Annexin-V/7-AAD (FACS strategy 1). **c.** Colony-forming units of 300 scrambled and IFNγR1^{KO} cells treated with Ruxo, cultured for 7 days, and stained with crystal violet. Experiments in **a-c** were repeated thrice with similar results. **d-i.** B6 mice bearing IFNγR1^{KO} melanoma were treated with vehicle (UnTx) or with Ruxo (90 mg/kg by oral gavage twice daily for 10 days). Isolated tumor cells ($CD45^-$) were analyzed for p-JAK2 (*, p=0.0416) and p-STAT3 (*, p=0.0171) (**d**) (n=4 per group). Isolated splenocytes were analyzed for frequencies of $FoxP3^+$ T_{reg} (**e**) (****, p=0.00008) and of $CD4^+$ and $CD8^+$ T cells (**i**). Isolated TILs were analyzed for IFN-γ (**f**) (*, p=0.0314), perforin (**g**) (**, p=0.0072), and GzmB (**h**) (*, p=0.0118) production by $CD8^+$ TILs after a brief stimulation with PMA and ionomycin. FACS strategy 2 was used for **e** and **i**; FACS strategy 3 was applied in **d** and **f-h.** N=6 for UnTx and n=7 for Ruxo groups in **e-i**. Results in the scatter plots depict means ± SEM. Two-sided Student's t-test was used for all statistical analyses. Source data are provided as a Source Data file.

Supplementary Figure 7. Ruxo suppression of IFNγR1^{KO} melanomas relies on host TNF.

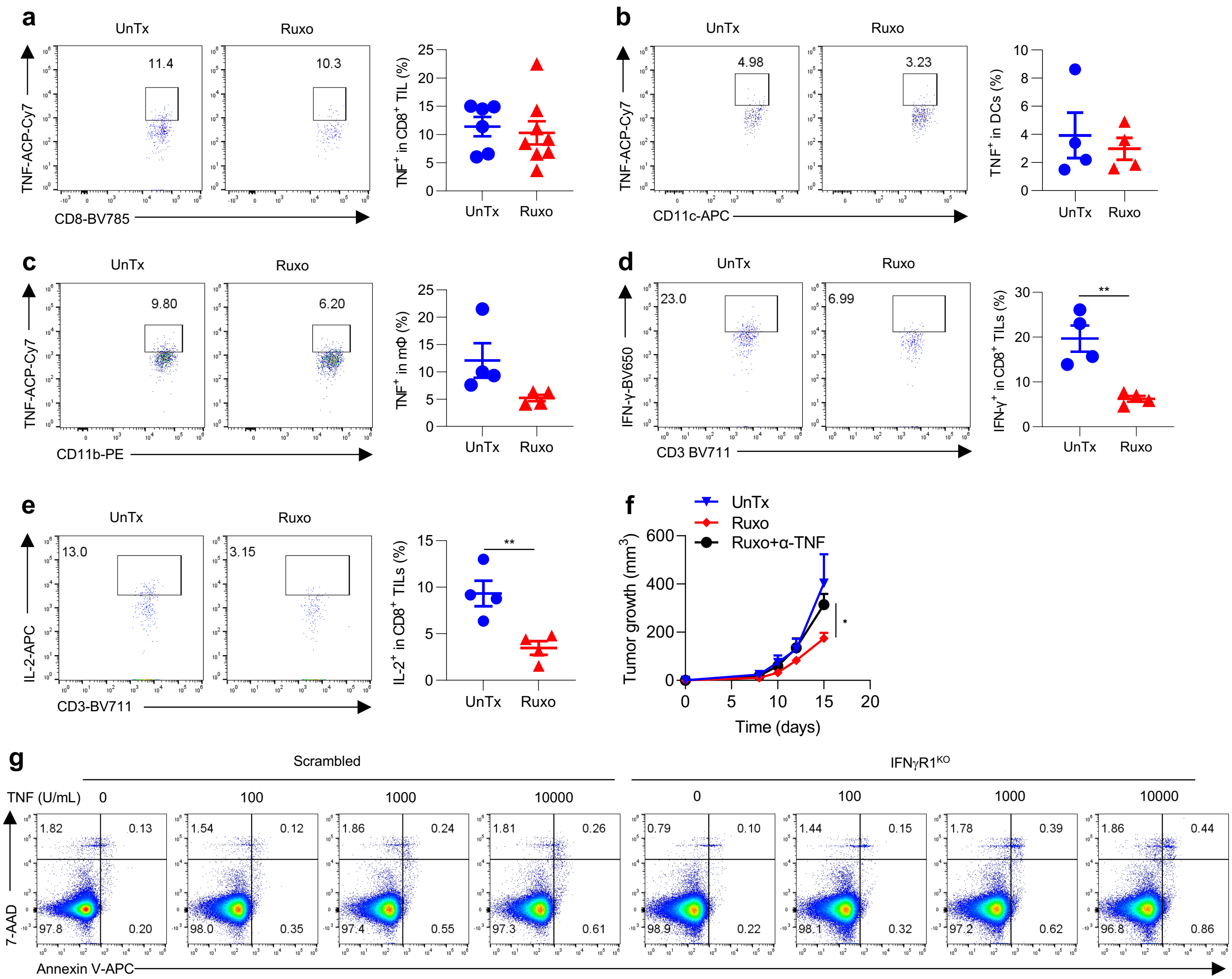

a-c. B6 mice bearing IFNγR1^{KO} melanoma were treated with vehicle (UnTx) or Ruxo. TNF production by CD8^{+} TILs (**a**), dendritic cells (**b**) and macrophages (**c**) were shown, after a brief stimulation with PMA and ionomycin. **d-e.** CD8^{+} TILs from TNF$^{-/-}$ mice treated with vehicle (UnTx) and Ruxo were assessed for production of IFN-γ (**d**) (**, p=0.0041) and IL-2 (**e**) (**, p=0.0093) after a brief stimulation with PMA and ionomycin. **f.** Growth of IFNγR1^{KO} melanoma in B6 mice treated with vehicle (UnTx) (n=5), Ruxo (n=5), and Ruxo+α-TNF (n=5). α-TNF antibodies were given one day prior to Ruxo, repeated every 3 days. *, p=0.0319 by two-way ANOVA with Tukey's multiple comparisons test with adjustment. **g.** Scrambled control and IFNγR1^{KO} cells were treated with various concentrations of TNF for 96h, followed by Annexin-V/7-AAD staining to assess cell apoptosis (FACS strategy 1). Representative results from 3 independent experiments were shown. FACS strategy 3 was applied in **a-e.** N=6 for UnTx and n=7 for Ruxo in **a**. N=4 per group for **b-e**. Data in scatter plots and line graphs depict means ± SEM. Two-sided Student's t-test was used for all statistical analyses unless otherwise stated. Source data are provided as a Source Data file.

Supplementary Figure 8. FACS sequential gating/sorting strategies

FACS strategy 1: for cultured tumor cells.

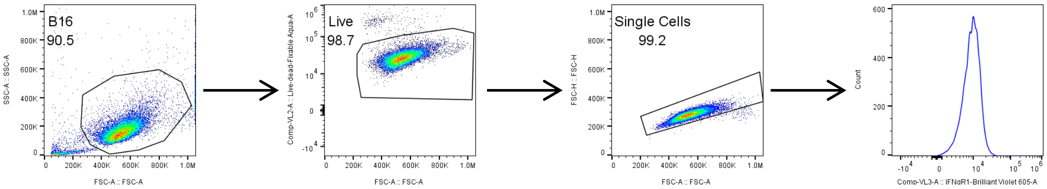

FACS strategy 2: for splenocytes.

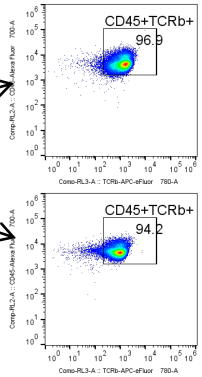
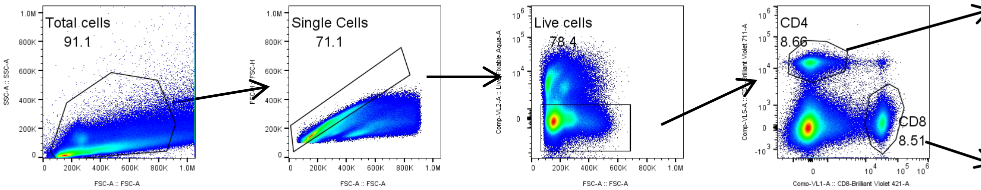

FACS strategy 3. for single cell suspensions of tumors.

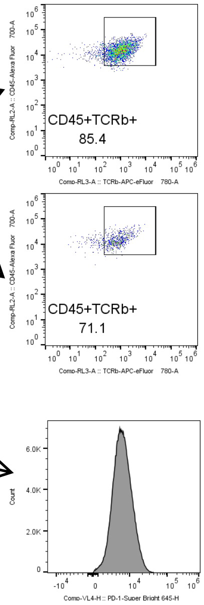
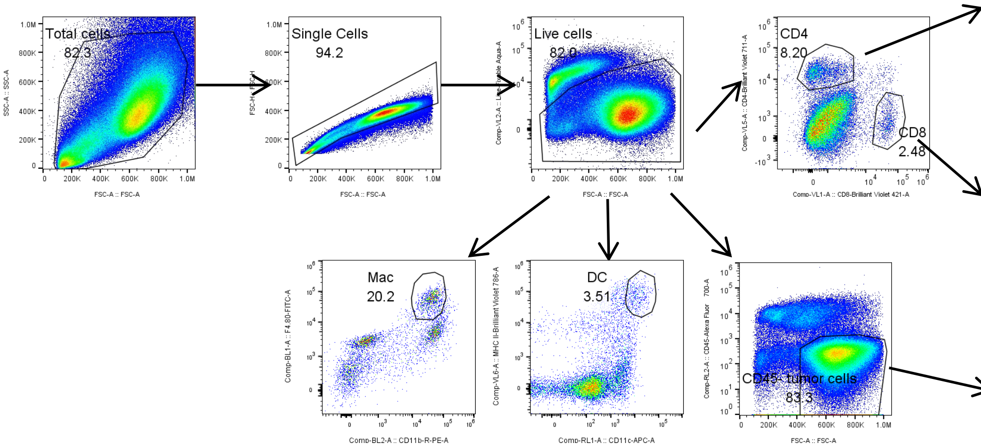

Supplementary Figure 9. Uncropped scans of blots-Set 1

Fig. S3a

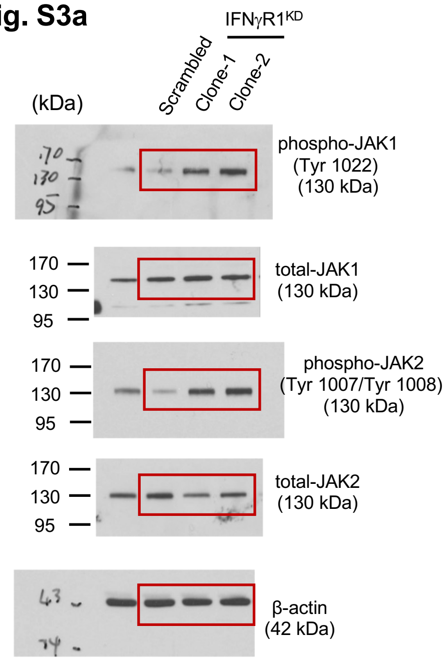

Fig. S3b

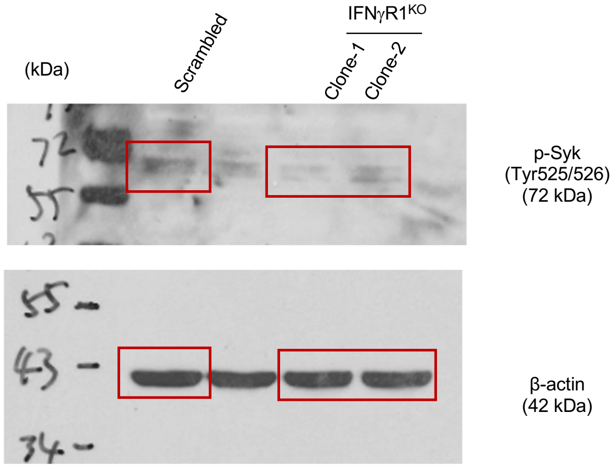

Fig. S3c

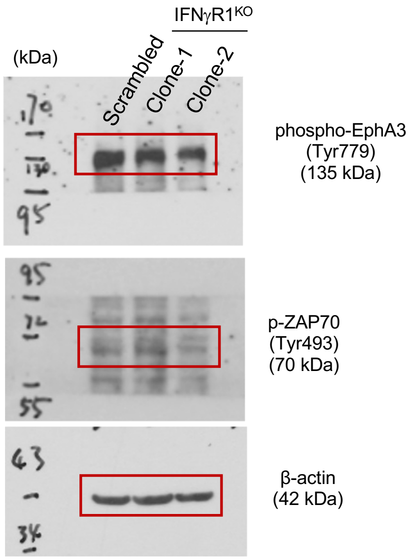

Supplementary Figure 9. Uncropped scans of blots-Set 2

Fig. S4a

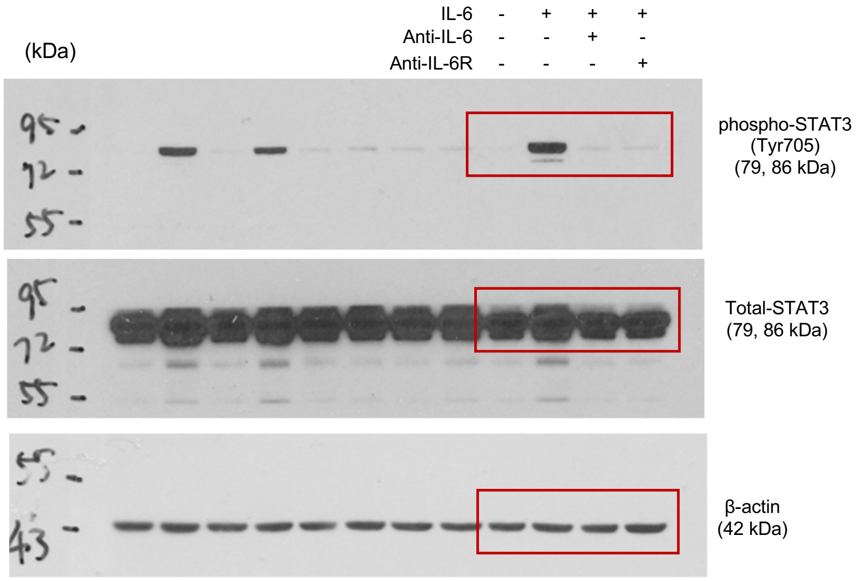

Fig. S4b

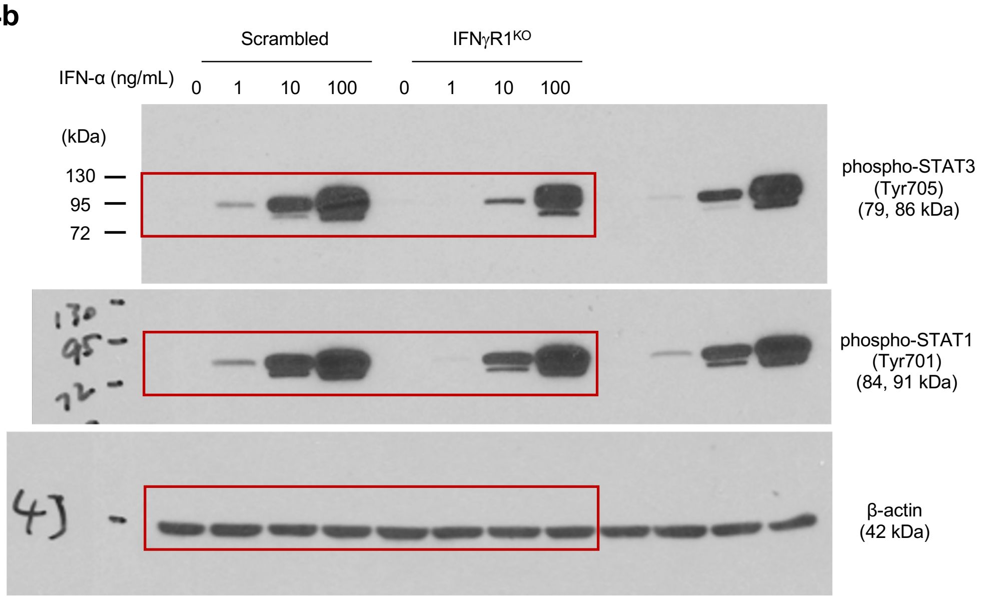

Supplementary Figure 9. Uncropped scans of blots-Set 3

Fig. S5a

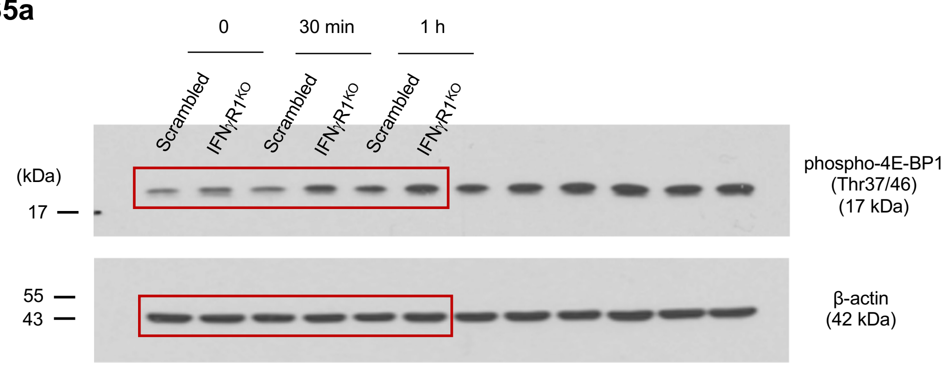

Fig. S6a

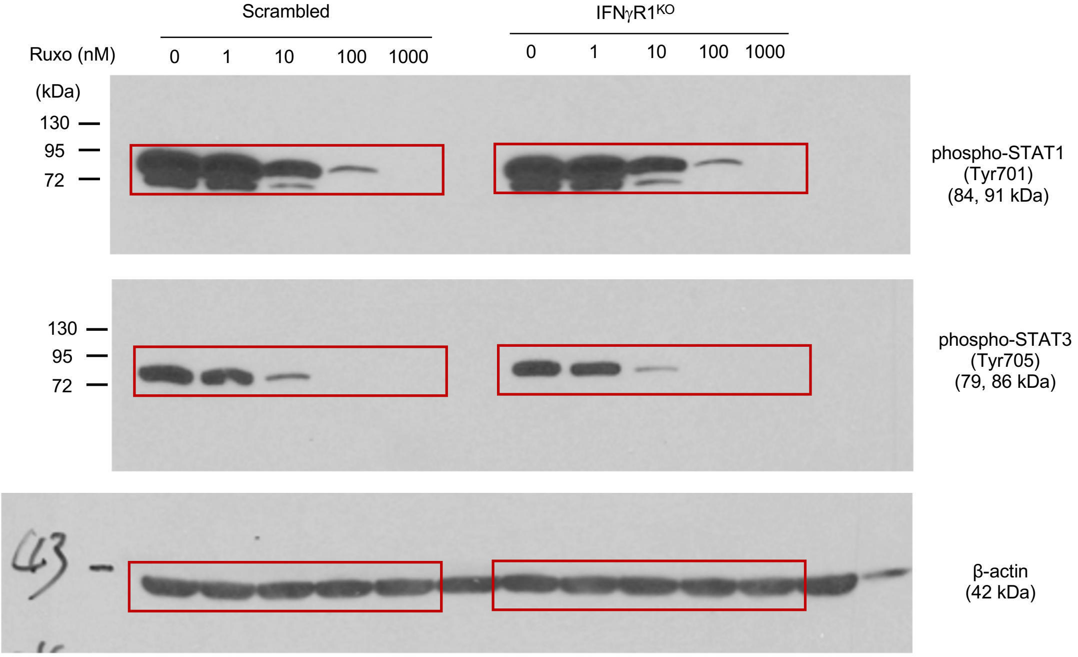

Supplementary Table 1. Constitutively active protein tyrosine kinases in IFNγR1^{KO} cells

Kinase Uniprot ID	Kinase Name	Mean Final Score	Mean Kinase Statistic
P43405	Syk	1.928705245	0.54728096
P43403	ZAP70	1.382603379	0.527385799
P29320	EphA3	1.20429617	0.883373601
P23458	JAK1	1.173362264	0.920409894
P54760	EphB4	0.934701591	0.80521969
P54756	EphA5	0.918173102	0.76725716
Q15375	EphA7	0.899031478	0.793955326
P09619	PDGFRb	0.878173195	0.628206115
P21709	EphA1	0.846162673	0.692038084
P10721	Kit	0.78207578	0.600426181
P22455	FGFR4	0.776790182	0.558136724
P29323	EphB2	0.757309665	0.720546186
Q6ZMQ8	Lmr1	0.723290296	0.667899052
P08631	HCK	0.71456302	0.534514856
P51451	BLK	0.711316513	0.523465992
P12931	Src	0.698001931	0.502406768
Q06418	Tyro3/Sky	0.696794544	0.497390036
O60674	JAK2	0.656632684	0.08439117
P29597	Tyk2	0.645893531	0.603603184
P14616	IRR	0.63890575	0.558754681
P54753	EphB3	0.585026652	0.580896437
P04629	TRKA	0.581015208	0.527320494
P29376	LTK	0.575013598	0.526519256
P06241	Fyn	0.574084042	0.533498414
Q13882	Brk	0.5260817	0.459873612
H0Y8A4	RYK	0.522939044	0.529884982

Supplementary Table 2. Activated signaling pathways in IFNγR1^{KO} cells.

Identified signaling pathways	Kinases in each pathway	Increased phosphorylation sites																			Count
		Cad-pS1859	Eif4g1-pS1187	Eps8-pT628	Hmga1-pS103	Larp1-pS743	Lyn-pY508	Map1b-pS1247	Mapt-pS688	Mapt-pS692	Rangap1-pS444	Rps3-pT221	Rrm2-pS20	Sort1-pS819	Stub1-pS20	Stx7-pS125	Stx7-pS126	Tln1-pS425	2-Sep-pS218	U2af2-pS79	
		PRKACA, Rps6kb1	Prkca	Mapk3	Csnk2a1	mTOR	CSK,Matk,LYN	mTOR	Prkaa1,GSK3B,Mapk3,Csnk1d,GSK3A,Prkca,MARK1,Mapk12,Mapk1,DYRK1A,CDK5,CAMK2A,Mapk13,Mapk8,TTBK1	GSK3A,GSK3B	CDK1, CDK2	Prkcd,CDK1, Mapk1	CDK1	Csnk2b	CDK5	Prkca	Akt1	CDK5	CK2A1	CDK1	
**PI3K-Akt signaling pathway**	Akt1; Cdk2; Gsk3b; Mapk1; Mapk3; Mtor; Prkaa1; Prkca; Rps6kb1	X	X	X		X		X	X	X	X	X				X	X				11
**Growth hormone synthesis**	Akt1; Gsk3b; Mapk1; Mapk12; Mapk13; Mapk3; Mapk8; Mtor; Prkaca; Prkca	X	X	X		X		X	X	X		X				X	X				10
**ErbB signaling pathway**	Akt1; Camk2a; Gsk3b; Mapk1; Mapk3; Mapk8; Mtor; Prkca; Rps6kb1	X	X	X		X		X	X	X		X				X	X				10
**mTOR signaling pathway**	Akt1; Gsk3b; Mapk1; Mapk3; Mtor; Prkaa1; Prkca; Rps6kb1	X	X	X		X		X	X	X		X				X	X				10
**EGFR tyrosine kinase inhibitor resistance**	Akt1; Gsk3b; Mapk1; Mapk3; Mtor; Prkca; Rps6kb1	X	X	X		X		X	X	X		X				X	X				10
**Cellular senescence**	Akt1; Cdk1; Cdk2; Mapk1; Mapk12; Mapk13; Mapk3; Mtor			X		X		X	X		X	X	X				X			X	9
**Insulin signaling pathway**	Akt1; Gsk3b; Mapk1; Mapk3; Mapk8; Mtor; Prkaa1; Prkaca; Rps6kb1	X		X		X		X	X	X		X					X				8
**Autophagy**	Akt1; Dapk1; Dapk3; Mapk1; Mapk3; Mapk8; Mtor; Prkaa1; Prkaca; Prkcd; Rps6kb1	X		X		X		X	X			X					X				7
**Focal adhesion**	Akt1; Gsk3b; Ilk; Mapk1; Mapk3; Mapk8; Prkca; Rock1; Rock2		X	X					X	X		X				X	X				7
**Endocrine resistance**	Akt1; Mapk1; Mapk12; Mapk13; Mapk3; Mapk8; Mtor; Prkaca; Rps6kb1	X		X		X		X	X			X					X				7
**Relaxin signaling pathway**	Akt1; Prkaca; Prkca; Mapk1; Mapk13; Mapk3; Mapk8; Mapk12	X	X	X					X			X				X	X				7
**Wnt signaling pathway**	Camk2a; Csnk2a1; Csnk2b; Prkaca; Prkca; Rock2; Mapk8; Gsk3b	X	X		X				X	X				X		X					7
**MAPK signaling pathway**	Akt1; Mapk1; Mapk12; Mapk13; Mapk3; Mapk8; Prkaca; Prkca	X	X	X					X			X				X	X				7
**PD-1/L1 pathway in cancer**	Akt1; Csnk2a1; Csnk2b; Mapk1; Mapk3; mTOR; Rps6kb	X		X	X	X		X	X			X									7
**Chemokine signaling pathway**	Akt1; Gsk3a; Gsk3b; Lyn; Mapk1; Mapk3; Prkaca; Prkcd; Rock1; Rock2	X		X					X	X		X					X				6
**GnRH signaling pathway**	Camk2a; Mapk1; Mapk12 Mapk13; Mapk3; Mapk8; Prkaca; Prkca; Prkcd	X	X	X					X			X				X					6
**VEGF signaling pathway**	Akt1; Mapk1; Mapk12; Mapk13; Mapk3; Prkca		X	X					X			X				X	X				6
**cAMP signaling pathway**	Akt1; Camk2a; Mapk1; Mapk3; Mapk8; Prkaca; Rock1; Rock2	X		X					X			X					X				5
**FoxO signaling pathway**	Akt1; Cdk2; Mapk1; Mapk12; Mapk13; Mapk3; Mapk8; Prkaa1			X					X		X	X					X				5
**AMPK signaling pathway**	Akt1; Mtor; Prkaa1; Rps6kb1	X				X		X	X								X				5
**TGF-beta signaling pathway**	Mapk1; Mapk3; Rock1; Rps6kb1	X		X					X			X									4
**TNF signaling pathway**	Akt1; Mapk1; Mapk12; Mapk13 Mapk3; Mapk8			X					X			X					X				4
**IL-17 signaling pathway**	Gsk3b; Mapk1; Mapk12; Mapk13; Mapk3; Mapk8			X					X	X		X									4

Note: Cell lysates were analyzed by phosphoproteomics. Increased phosphorylation sites of the defined proteins and corresponding kinases catalyzing these phosphorylations were listed in the table. Counts indicate how many of the identified 19 phosphorylation sites are mediated by the kinases in each signaling pathway. The higher the count is, the greater probability of the activation of that pathway is.
