# Supplementary material for: Selective suppression of melanoma lacking IFN-γ pathway by JAK inhibition depends on T cells and host TNF signaling
Source: Nat Commun. 2022 Aug 25;13:5013. doi: 10.1038/s41467-022-32754-7 (PMC9411168; doi:10.1038/s41467-022-32754-7)
Supplement: Supplementary file 3 — Reporting Summary [file 41467_2022_32754_MOESM3_ESM.pdf]

nature portfolio

Corresponding author(s): Lewis Z. Shi; James A. Bonner; Zechen Chong

Last updated by author(s): Aug 6, 2022

Reporting Summary

Nature Portfolio wishes to improve the reproducibility of the work that we publish. This form provides structure for consistency and transparency in reporting. For further information on Nature Portfolio policies, see our Editorial Policies and the Editorial Policy Checklist.

Statistics

For all statistical analyses, confirm that the following items are present in the figure legend, table legend, main text, or Methods section.

n/a	Confirmed	
☐	☒	The exact sample size (*n*) for each experimental group/condition, given as a discrete number and unit of measurement
☐	☒	A statement on whether measurements were taken from distinct samples or whether the same sample was measured repeatedly
☐	☒	The statistical test(s) used AND whether they are one- or two-sided<br>*Only common tests should be described solely by name; describe more complex techniques in the Methods section.*
☐	☒	A description of all covariates tested
☐	☒	A description of any assumptions or corrections, such as tests of normality and adjustment for multiple comparisons
☐	☒	A full description of the statistical parameters including central tendency (e.g. means) or other basic estimates (e.g. regression coefficient) AND variation (e.g. standard deviation) or associated estimates of uncertainty (e.g. confidence intervals)
☐	☒	For null hypothesis testing, the test statistic (e.g. *F*, *t*, *r*) with confidence intervals, effect sizes, degrees of freedom and *P* value noted<br>*Give P values as exact values whenever suitable.*
☒	☐	For Bayesian analysis, information on the choice of priors and Markov chain Monte Carlo settings
☒	☐	For hierarchical and complex designs, identification of the appropriate level for tests and full reporting of outcomes
☒	☐	Estimates of effect sizes (e.g. Cohen's *d*, Pearson's *r*), indicating how they were calculated

Our web collection on statistics for biologists contains articles on many of the points above.

Software and code

Policy information about availability of computer code

Data collection	1. All the flow cytometric data were acquired using the built-in software of the Attune NxT Flow Cytometer (Invitrogen, A24860) from Thermo Fisher.<br>2. All the animal studies were conducted in an IACUC-accredited animal facility at UAB and tumor growth data were taken by regular measurements with a caliper.<br>3. All the Western blot results were obtained in the conventional film exposure methods in the dark room.<br>4. Kinomic profiling was performed in the UAB Kinome Core using chips for tyrosine kinases.<br>5. Phosphoproteomic data were acquired using SPS-MS3 approach with the Orbitrap Lumos mass spectrometer.
Data analysis	1. Flow cytometric data were analyzed using FlowJo version 10.8.1.<br>2. Raw image analysis of kinomic data was conducted using Evolve2, and comparative analysis of kinases upstream of altered peptide prediction was done in BioNavigator v6.3 using PTK and STK UpKin PamApps (v 6.0).<br>3. Phosphoproteomic hits were searched against human or mouse protein databases that were downloaded from uniprot.org, using the MaxQuant software.<br>4. All the statistical analyses of the data were done using Prism-GraphPad version 9.4.0.<br>5. For RNA-seq data analysis, paired-end transcriptome sequences were mapped to the Mus musculus GRCm38 reference genome available on ENSEMBL using the STAR aligner (version 2.7.5a). Read counts per gene were calculated using htseq-count in the HTseq package (version 0.11.2). The analysis of differentially expressed genes (DEGs) between the scrambled control and IFNgR1KO samples was performed using DESeq2 (version 1.34.0) in R (version 3.6.0). A volcano plot was used to show all upregulated and downregulated DEGs using the ggplot2 package (version 3.3.6). Enriched Kyoto Encyclopedia of Genes and Genomes (KEGG) pathways of the DEGs were identified by enrichr package (version 3.0)

For manuscripts utilizing custom algorithms or software that are central to the research but not yet described in published literature, software must be made available to editors and reviewers. We strongly encourage code deposition in a community repository (e.g. GitHub). See the Nature Portfolio guidelines for submitting code & software for further information.

Data

Policy information about availability of data

All manuscripts must include a data availability statement. This statement should provide the following information, where applicable:

- Accession codes, unique identifiers, or web links for publicly available datasets
- A description of any restrictions on data availability
- For clinical datasets or third party data, please ensure that the statement adheres to our policy

The publicly available skin cutaneous melanoma and uveal melanoma TCGA data used in this study are available in National Cancer Institute Genomics Data Commons (GDC) [https://gdc.cancer.gov/about-data/publications/pancanatlas]. The publicly available gene expression profiles of published pre-treatment melanomas undergoing anti-PD-1 therapy transcriptome data used in this study are available in the GEO database under accession code GSE78220. The RNA-seq data generated in this study have been deposited in the Gene Expression Omnibus (GEO) database under accession code GSE201078. The phosphoproteomic data generated in his study have been deposited in the Mass Spectrometry Interactive Virtual Environment (MassIVE) database under accession ID MSV000087796. The authors made step-by-step instructions on how to access this proteomic dataset in the Supplemental Information file.

Field-specific reporting

Please select the one below that is the best fit for your research. If you are not sure, read the appropriate sections before making your selection.

[x] Life sciences [ ] Behavioural & social sciences [ ] Ecological, evolutionary & environmental sciences

For a reference copy of the document with all sections, see nature.com/documents/nr-reporting-summary-flat.pdf

Life sciences study design

All studies must disclose on these points even when the disclosure is negative.

Sample size	We decide sample size based on our working experiences in this field, as reported in our previous publications (PMID: 27498556, PMID: 27667683, and PMID: 31466995). For in vitro, the minimum of 3 independent samples were used. For in vivo, 5 mice per group were anticipated in most of the experiments.
Data exclusions	All the animals were housed in animal facility for one week before tumor inoculation to exclude any unhealthy mice.
Replication	All the animal experiments were done at least twice, and each group had at least 5 mice to start with, although most of them were done for more than 3 times (as indicated). All the in vitro experiments were independently repeated, with similar results. All attempts at replication were successful.
Randomization	Animals were randomly allocated to groups with a table of random numbers.
Blinding	To avoid observer-expectation bias, tumor inoculation, tumor size measurement, and final data analysis were performed by different people.

Reporting for specific materials, systems and methods

We require information from authors about some types of materials, experimental systems and methods used in many studies. Here, indicate whether each material, system or method listed is relevant to your study. If you are not sure if a list item applies to your research, read the appropriate section before selecting a response.

Materials & experimental systems

n/a	Involved in the study	
[ ]	[x]	Antibodies
[ ]	[x]	Eukaryotic cell lines
[x]	[ ]	Palaeontology and archaeology
[ ]	[x]	Animals and other organisms
[x]	[ ]	Human research participants
[x]	[ ]	Clinical data
[x]	[ ]	Dual use research of concern

Methods

n/a	Involved in the study	
[x]	[ ]	ChIP-seq
[ ]	[x]	Flow cytometry
[x]	[ ]	MRI-based neuroimaging

Antibodies

Antibodies used	Flow antibodies used: Aqua fixation LIVE/DEAD™ Fixable Aqua Dead Cell Stain Kit (1:200, Thermo Fisher, #L34966), CD4-BV421 (1:200, clone RM4-5, BioLegend, #100544), CD8-BV786 (1:200, clone 53-6.7, BD Biosciences, #563332), CD45- PerCP-Cyanine5.5 (1:200, clone 30-F11, Thermo Fisher, #45-0451-82), CD11b-PE (1:200, clone M1/70, Biolegend, #101208), CD11c-APC (1:200, clone

N418, Biolegend, #117310), F4/80-BV785 (1:200, clone BM8, Biolegend, #123141), TCRβ-APC Cy7 (1:200, clone H57-597, Biolegend, #109220), CD3-BV711 (1:200, clone 145-2C11, Biolegend, #100349), IFNγR1-BV605 (1:200, clone GR20, BD Biosciences, #745111), IFNαR1-APC (1:200, clone MAR1-5A3, Biolegend, #127313), PD-L1-APC (1:200, clone 10F.9G2, Biolegend, #124312), MHC I-BV650 (1:200, clone SF1-1.1, BD Biosciences, #742434), MHC II-BV785 (1:200, clone M5/114.15.2, Biolegend, #107645), FoxP3-eFluor™ 450 (1:100, clone FJK-16s, Thermo Fisher, #48-5773-82), Perforin-PE (1:100, clone S16009A, Biolegend, #154306), TNF-APC Cy7 (1:100, MP6-XT22, Biolegend, #506344), PD-1-APC (1:100, clone RMP1-30, Thermo Fisher, # 17-9981-82), CD73-BV605 (1:200, clone TY/11.8, Biolegend, #127215), Granzyme B-FITC (1:100, clone QA16A02, Biolegend, #372206), IFN-γ-BV650 (1:100, clone XMG1.2, Biolegend, #505832), IL-2-BV711 (1:100, clone JES6-5H4, Biolegend, #503837), phospho-JAK2 (Tyr 1007/Tyr 1008)-APC (1:100, clone E132, Abcam, #ab200340) and phosphor-STAT3 (Tyr705)-FITC (1:100, clone LUVNKLA, Thermo Fisher, #11-9033-42).

The antibodies used for western blot are: phospho-JAK1 (1:1000, Santa Cruz Biotechnology, polyclonal, #sc-101716), total-JAK1 (1:1000, Santa Cruz Biotechnology, clone HR-785, #sc-277), phospho-JAK2 (1:1000, Santa Cruz Biotechnology, polyclonal, #sc-16566-R), total-JAK2 (1:1000, Santa Cruz Biotechnology, clone C-10, #sc-390539), phospho-AKT (Ser473) (1:1000, Cell Signaling Technology, polyclonal, #9271), total-AKT (1:1000, Cell Signaling Technology, polyclonal, #9272), phospho-4EBP1 (Thr37/46) (1:5000, Cell Signaling Technology, clone 236B4, #2855), phospho-STAT1 (Tyr701) (1:1000, Cell Signaling Technology, clone 58D6, #9167), total-STAT1 (1:1000, Cell Signaling Technology, polyclonal, #9172), phospho-STAT3 (Tyr705) (1:1000, Cell Signaling Technology, D3A7, #9145), total-STAT3 (1:1000, Cell Signaling Technology, 79D7, #4904), phospho-Syk (Tyr525/526) (1:1000, Cell Signaling Technology, C87C1, #2710), phospho-ZAP70 (Tyr493) (1:1000, Cell Signaling Technology, polyclonal, #2704T), phospho-EphA3 (Tyr779) (1:1000, Cell Signaling Technology, D10H1, #8862S), mTOR (1:1000, Cell Signaling Technology, clone 7C10, #2983) and β-actin (1:10000, Santa Cruz Biotechnology, #sc-47778 HRP).

Validation

We only order antibodies from highly-coveted reputable vendors (see above description) and all the antibodies that we used have been well-validated by the vendors, with validation statements available on manufacturer's websites, and commonly used in other studies. Flow antibodies used:

Aqua fixation LIVE/DEAD™ Fixable Aqua Dead Cell Stain Kit (1:200, Thermo Fisher, #L34966. Use: Flow Cytometry) https://www.thermofisher.com/order/catalog/product/L34966?ef_id=Cj0KCQjwuaiXBhCCARIsAKZLt3mXTkuQN99VakwhA6uZtJ6Ii86QHd0nh5upfcWDC0BgNnRNUMvURfQaAkdjEALw_wcB:G:s&s_kwcid=AL!3652!3!601175229291!!!g!!!17329199729!133905464141&cid=bid_pca_frg_r01_co_cp1359_pjt0000_bid00000_0se_gaw_dy_pur_con&gclid=Cj0KCQjwuaiXBhCCARIsAKZLt3mXTkuQN99VakwhA6uZtJ6Ii86QHd0nh5upfcWDC0BgNnRNUMvURfQaAkdjEALw_wcB,

CD4-BV421 (1:200, clone RM4-5, BioLegend, #100544. Species reactivity: mouse. Use: Flow Cytometry.) https://www.biolegend.com/en-us/products/brilliant-violet-421-anti-mouse-cd4-antibody-7349,

CD8-BV786 (1:200, clone 53-6.7, BD Biosciences, #563332. Species reactivity: mouse. Use: Flow Cytometry) https://www.bdbiosciences.com/en-us/search-results?searchKey=563332,

CD45- PerCP-Cyanine5.5 (1:200, clone 30-F11, Thermo Fisher, #45-0451-82. Species reactivity: mouse. Use: Flow Cytometry) https://www.thermofisher.com/order/genome-database/dataSheetPdf?producttype=antibody&productsubtype=antibody_primary&productId=45-0451-82&version=243,

CD11b-PE (1:200, clone M1/70, Biolegend, #101208. Species reactivity: mouse, human. Use: Flow Cytometry, IHC) https://www.biolegend.com/en-us/products/pe-anti-mouse-human-cd11b-antibody-349,

CD11c-APC (1:200, clone N418, Biolegend, #117310. Species reactivity: mouse. Use: Flow Cytometry) https://www.biolegend.com/en-us/products/apc-anti-mouse-cd11c-antibody-1813,

F4/80-BV785 (1:200, clone BM8, Biolegend, #123141. Species reactivity: mouse. Use: Flow Cytometry) https://www.biolegend.com/en-us/products/brilliant-violet-785-anti-mouse-f4-80-antibody-9919,

TCRβ-APC Cy7 (1:200, clone H57-597, Biolegend, #109220. Species reactivity: mouse. Use: Flow Cytometry) https://www.biolegend.com/en-us/products/apc-cyanine7-anti-mouse-tcr-beta-chain-antibody-4137,

CD3-BV711 (1:200, clone 145-2C11, Biolegend, #100349. Species reactivity: mouse. Use: Flow Cytometry) https://www.biolegend.com/en-us/products/brilliant-violet-711-anti-mouse-cd3epsilon-antibody-11975,

IFNγR1-BV605 (1:200, clone GR20, BD Biosciences, #745111. Species reactivity: mouse. Use: Flow Cytometry) https://www.bdbiosciences.com/en-us/search-results?searchKey=745111,

IFNαR1-APC (1:200, clone MAR1-5A3, Biolegend, #127313. Species reactivity: mouse. Use: Flow Cytometry) https://www.biolegend.com/en-us/products/apc-anti-mouse-ifnar-1-antibody-4785,

PD-L1-APC (1:200, clone 10F.9G2, Biolegend, #124312. Species reactivity: mouse. Use: Flow Cytometry) https://www.biolegend.com/en-us/products/apc-anti-mouse-cd274-b7-h1-pd-l1-antibody-6655,

MHC I-BV650 (1:200, clone SF1-1.1, BD Biosciences, #742434. Species reactivity: mouse. Use: Flow Cytometry) https://www.bdbiosciences.com/en-us/products/reagents/flow-cytometry-reagents/research-reagents/single-color-antibodies-ruo/bv650-mouse-anti-mouse-h-2kd.742434,

MHC II-BV785 (1:200, clone M5/114.15.2, Biolegend, #107645. Species reactivity: mouse. Use: Flow Cytometry) https://www.biolegend.com/en-us/products/brilliant-violet-785-anti-mouse-i-a-i-e-antibody-12087,

FoxP3-eFluor™ 450 (1:100, clone FJK-16s, Thermo Fisher, #48-5773-82. Species reactivity: Rat, Bovine, Dog, Cat, Mouse, Pig. Use: Flow Cytometry) https://www.thermofisher.com/antibody/product/FOXP3-Antibody-clone-FJK-16s-Monoclonal/48-5773-82,

Perforin-PE (1:100, clone S16009A, Biolegend, #154306. Species reactivity: mouse. Use: Flow Cytometry) https://www.biolegend.com/en-us/products/pe-anti-mouse-perforin-antibody-15255,

TNF-APC Cy7 (1:100, MP6-XT22, Biolegend, #506344. Species reactivity: mouse. Use: Flow Cytometry) https://www.biolegend.com/en-us/products/apc-cyanine7-anti-mouse-tnf-alpha-antibody-12117,

PD-1-APC (1:100, clone RMP1-30, Thermo Fisher, # 17-9981-82. Species reactivity: mouse. Use: Flow Cytometry) https://www.thermofisher.com/antibody/product/CD279-PD-1-Antibody-clone-RMP1-30-Monoclonal/17-9981-82,

CD73-BV605 (1:200, clone TY/11.8, Biolegend, #127215. Species reactivity: mouse. Use: Flow Cytometry) https://www.biolegend.com/en-us/products/brilliant-violet-605-anti-mouse-cd73-antibody-8153,

Granzyme B-FITC (1:100, clone QA16A02, Biolegend, #372206. Species reactivity: mouse, human. Use: Flow Cytometry) https://www.biolegend.com/en-us/products/fitc-anti-human-mouse-granzyme-b-recombinant-antibody-14430,

IFN-γ-BV650 (1:100, clone XMG1.2, Biolegend, #505832. Species reactivity: mouse. Use: Flow Cytometry) https://www.biolegend.com/en-us/products/brilliant-violet-650-anti-mouse-ifn-gamma-antibody-7681,

IL-2-BV711 (1:100, clone JES6-5H4, Biolegend, #503837. Species reactivity: mouse. Use: Flow Cytometry) https://www.biolegend.com/en-us/products/brilliant-violet-711-anti-mouse-il-2-antibody-10339,

phospho-JAK2 (Tyr 1007/Tyr 1008)-APC (Tyr 1007/Tyr 1008)-APC (1:100, clone E132, Abcam, #ab200340. Species reactivity: human, mouse, rat. Use: Flow Cytometry, IF) https://www.abcam.com/alexa-fluor-647-jak2-phospho-y1007--y1008-antibody-e132-ab200340.html,
phosphor-STAT3 (Tyr705)-FITC (1:100, clone LUVNKLA, Thermo Fisher, #11-9033-42. Species reactivity: human, mouse. Use: Flow Cytometry)https://www.thermofisher.com/antibody/product/Phospho-STAT3-Tyr705-Antibody-clone-LUVNKLA-Monoclonal/11-9033-42,

The antibodies used for western blot are:
phospho-JAK1 (1:1000, Santa Cruz Biotechnology, polyclonal, #sc-101716. Species reactivity: human, mouse, rat. Use: WB, IF, IHC, IP) https://www.citeab.com/antibodies/3430240-sc-101716-p-jak1-tyr-1022,
total-JAK1 (1:1000, Santa Cruz Biotechnology, clone HR-785, #sc-277. Species reactivity: human, mouse. Use: WB, IHC, IP) https://www.citeab.com/antibodies/799720-sc-277-jak1-antibody-hr-785,
phospho-JAK2 (1:1000, Santa Cruz Biotechnology, polyclonal, #sc-16566-R. Species reactivity: human, rat. Use: WB) https://www.citeab.com/antibodies/3430242-sc-16566-r-p-jak2-tyr-1007-tyr-1008-r,
total-JAK2 (1:1000, Santa Cruz Biotechnology, clone C-10, #sc-390539. Species reactivity: human, mouse, rat. Use: WB, IF, IHC, IP) https://www.scbt.com/p/jak2-antibody-c-10,
phospho-AKT (Ser473) (1:1000, Cell Signaling Technology, polyclonal, #9271. Species reactivity: human, mouse, rat. Use: WB, IF, IHC, IP) https://www.cellsignal.com/products/primary-antibodies/phospho-akt-ser473-antibody/9271,
total-AKT (1:1000, Cell Signaling Technology, polyclonal, #9272. Species reactivity: human, mouse, rat, Mk, Dog. Use: WB, IF, ChIP, Flow cytometry) https://www.cellsignal.com/products/primary-antibodies/akt-antibody/9272,
phospho-4EBP1 (1:5000, Cell Signaling Technology, clone 236B4, #2855. Species reactivity: human, mouse, rat, Mk, Dog. Use: WB, IF, ChIP, Flow cytometry) https://www.cellsignal.com/products/primary-antibodies/phospho-4e-bp1-thr37-46-236b4-rabbit-mab/2855,
phospho-STAT1 (Tyr701) (1:1000, Cell Signaling Technology, clone 58D6, #9167. Species reactivity: human, mouse. Use: WB, IF, ChIP, Flow cytometry) https://www.cellsignal.com/products/primary-antibodies/phospho-stat1-tyr701-58d6-rabbit-mab/9167,
total-STAT1 (1:1000, Cell Signaling Technology, polyclonal, #9172. Species reactivity: human, mouse, rat, Mk, Dog, Rab. Use: WB, IF, ChIP, Flow cytometry) https://www.cellsignal.com/products/primary-antibodies/stat1-antibody/9172,
phospho-STAT3 (Tyr705) (1:1000, Cell Signaling Technology, D3A7, #9145. Species reactivity: human, mouse, rat, Mk. Use: WB, IF, ChIP, Flow cytometry) https://www.cellsignal.com/products/primary-antibodies/phospho-stat3-tyr705-d3a7-xp-rabbit-mab/9145,
total-STAT3 (1:1000, Cell Signaling Technology, 79D7, #4904. Species reactivity: human, mouse, rat, Mk, Dog, Rab. Use: WB, IF, ChIP, Flow cytometry) https://www.cellsignal.com/products/primary-antibodies/stat3-79d7-rabbit-mab/4904,
phospho-Syk (Tyr525/526) (1:1000, Cell Signaling Technology, C87C1, #2710. Species reactivity: human. Use: WB, IF, ChIP, Flow cytometry) https://www.cellsignal.com/products/primary-antibodies/phospho-syk-tyr525-526-c87c1-rabbit-mab/2710,
phospho-ZAP70 (Tyr493) (1:1000, Cell Signaling Technology, polyclonal, #2704. Species reactivity: human. Use: WB, IF, ChIP, Flow cytometry) https://www.cellsignal.com/products/primary-antibodies/phospho-zap-70-tyr493-syk-tyr526-antibody/2704,
phospho-EphA3 (Tyr779) (1:1000, Cell Signaling Technology, D10H1, #8862. Species reactivity: human, mouse, rat, Mk, Dog, Rab. Use: WB, IF, ChIP, Flow cytometry) https://www.cellsignal.com/products/primary-antibodies/phospho-epha3-tyr779-d10h1-rabbit-mab/8862,
mTOR (1:1000, Cell Signaling Technology, clone 7C10, #2983. Species reactivity: human, mouse, rat, Mk, Dog, Rab. Use: WB, IF, ChIP, Flow cytometry) https://www.cellsignal.com/products/primary-antibodies/mtor-7c10-rabbit-mab/2983,
β-actin (1:10000, Santa Cruz Biotechnology, #sc-47778 HRP . Species reactivity: human, mouse, rat, Rab. Use: WB, IF, ChIP, ELISA) https://www.scbt.com/p/beta-actin-antibody-c4?gclid=Cj0KCQjwuaiXBhCCARIsAKZLt3kvltZlETWCyIGQmhrV3rWipuw9YkR7quUZHmpwl5T-Ta2AvH4W48QaAuAeEALw_wcB.

Eukaryotic cell lines

Policy information about cell lines

Cell line source(s): The B16-BL6 murine melanoma cells were kindly provided by Dr I. Fidler at MD Anderson Cancer Center. B16-BL6 IFNgR1 KD and scrambled control cells were similarly maintained and used as we previously described (PMID: 27667683).

Authentication: All cell lines used are either from ATCC or commonly used by many investigators. Some of them were obtained from Dr. Jim Allison and Dr. Pam Sharma lab when I left MD Anderson Cancer Center to establish my own lab in 2017. All cell lines are not among the contamination list of the International Cell Line Authentication Committee.

Mycoplasma contamination: All cells are regularly tested and remain free of mycoplasma.

Commonly misidentified lines (See ICLAC register): We did not use any misidentified lines.

Animals and other organisms

Policy information about studies involving animals; ARRIVE guidelines recommended for reporting animal research

Laboratory animals: Seven-week-old female C57BL/6 (Stock No: 000664), Rag-1-/- (Stock No: 002216) and TNF-/- (Stock No: 005540) mice were purchased from The Jackson Laboratory (Bar Harbor, ME). All mice were housed in specific pathogen-free conditions in the animal facility of The University of Alabama at Birmingham (UAB) under 12 hours/12 hours light/dark cycle, ambient room temperature (22 ° C) with 40%-70% humidity.

Wild animals: We did not use wild animals in this study.

Field-collected samples: We did not have field-collected samples; all data were generated in my laboratory, from the public TCGA database, or from the

	published database (GSE78220).
Ethics oversight	All animal protocols were approved by Institutional Animal Care and Use Committee (IACUC) at UAB (APN-21945).

Note that full information on the approval of the study protocol must also be provided in the manuscript.

Flow Cytometry

Plots

Confirm that:

☒ The axis labels state the marker and fluorochrome used (e.g. CD4-FITC).

☒ The axis scales are clearly visible. Include numbers along axes only for bottom left plot of group (a 'group' is an analysis of identical markers).

☒ All plots are contour plots with outliers or pseudocolor plots.

☒ A numerical value for number of cells or percentage (with statistics) is provided.

Methodology

Sample preparation	Tumors were collected in ice-cold RPMI 1640 containing 2% FBS and minced into fine pieces, followed by digestion with 400 U/mL collagenase D (Worthington Biochemical Corporation, LS004186) and 20 μg/mL DNase I (Sigma, 10104159001) at 37 C for 40 min with periodic shaking. EDTA (Sigma, 1233508) was then added to the final concentration of 10 mM to stop digestion. Cell suspensions were filtered through 70 μM cell strainers, and TILs were obtained by collecting the cells in the interphase after Ficoll (MP Biomedicals, 091692254). Spleens were collected in ice-cold HBSS containing 2% FBS to prepare single cell suspensions, after lysis of red blood cells and filtering with nylon mesh. Both TILs and splenocytes were resuspended in complete Click's culture medium for flow cytometric analyses. In some experiments, isolated TILs were cultured with 100 U/mL IL-2, with or without 1 uM Ruxo for 3 days and analyzed for FoxP3 expression and production of IFN-g/TNF by flow cytometry. Surface staining of TILs and splenocytes was done in DPBS containing 2% BSA for 30 min on ice. To analyze FoxP3, following surface staining, cells were fixed using the Foxp3/Transcription Factor Staining Buffer Set (Invitrogen, 00-5523-00) and stained for FoxP3, according to the manufacturer's instructions. To detect intracellular cytokines, cells were briefly stimulated for 4-5 h with PMA (final concentration: 50 ng/mL) plus ionomycin (final concentration: 1M) in the presence of monensin (for the last 2 h). Stimulated cells were stained with surface markers, fixed using the BD Cytofix/Cytoperm Plus Fixation/Permeabilization Kit (BD Biosciences, 554715), and stained for cytokines, according to the manufacturer's instructions
Instrument	Attune NxT Flow Cytometer (Invitrogen, A24860)
Software	Data were acquired using the built-in acquisition software of Attune Flow Cytometer and analyzed using Flowjo software (Tree Star). Cell cycle distribution was determined using the cell cycle analysis function from Flowjo based on the Watson model.
Cell population abundance	The abundance of the relevant cell populations were presented in percentages, which were clearly defined using well-recognized markers.
Gating strategy	The preliminary FSC/SSC gates representing either total cells or lymphocytes were further gated on singlets (FSC-H vs FSC-A) and then live cells based on the staining with a fixable live/dead dye. The positive vs negative gating was based on no-stain controls (FMO) and the senior author's many years of working experiences in the field.

☒ Tick this box to confirm that a figure exemplifying the gating strategy is provided in the Supplementary Information.
